# Postoperative Urinary Retention After General Surgical Procedures: Incidence, Risk Factors, and Short-Term Outcomes

**DOI:** 10.7759/cureus.112178

**Published:** 2026-07-06

**Authors:** Prabhat Kumar N, Naveen Ch, Trisha C, Muralidhar Chinnapaka, Ramachakra Kushal Thota

**Affiliations:** 1 Internal Medicine, NIMRA Institute of Medical Sciences (NIMS), Vijayawada, IND; 2 General Surgery, Dr. Pinnamaneni Siddhartha Institute of Medical Sciences, Vijayawada, IND; 3 Internal Medicine, Chalmeda Anand Rao Institute of Medical Sciences, Karimnagar, IND; 4 Pharmacology, Government Medical College and Hospital Maheshwaram, Hyderabad, IND; 5 Internal Medicine, Gandhi Medical College, Secunderabad, IND

**Keywords:** bladder monitoring, general surgery, hospital stay, postoperative complications, postoperative urinary retention, pour, risk factors, spinal anaesthesia, urinary catheterisation, urinary tract infection

## Abstract

Background: Postoperative urinary retention (POUR) is a frequent but sometimes missed complication after general surgical procedures. If not recognized early, it may cause bladder overdistension, patient discomfort, urinary tract infection, unexpected catheterization, delayed mobilization and longer hospital stay. This study was conducted to determine the incidence, associated risk factors, and short-term outcomes of POUR in patients undergoing general surgical procedures.

Methods: This prospective observational study included adult patients who underwent elective or emergency general surgical procedures under spinal, regional, or general anesthesia. Patients with pre-existing urinary retention, chronic catheterization, neurogenic bladder, or known obstructive uropathy were excluded. POUR was defined as failure to void within six to eight hours after surgery, associated with suprapubic discomfort, bladder distension, or bladder volume requiring catheterization. Data regarding demographic profile, comorbidities, type and duration of surgery, anesthetic technique, perioperative intravenous fluid volume, postoperative opioid use, and early postoperative outcomes were recorded and analyzed.

Results: A total of 220 patients were included in the study. POUR developed in 32 patients (14.5%), while 188 patients (85.5%) did not develop urinary retention. POUR was more common in patients aged above 60 years (16/63 (25.4%)) than in those aged 60 years or below (16/157 (10.2%)). Male patients had a higher frequency of POUR (22/112 (19.6%)) compared with female patients (10/108 (9.3%)). Higher occurrence was also observed among patients with diabetes mellitus (12/50 (24.0%)), lower urinary tract symptoms (10/30 (33.3%)), spinal anesthesia (21/89 (23.6%)), operative duration >90 minutes (17/65 (26.2%)), perioperative intravenous fluid administration >1500 mL (20/83 (24.1%)) and postoperative opioid use (23/105 (21.9%)). Patients who developed POUR had higher rates of repeated catheterization, urinary tract infection, delayed mobilization, catheter-related discomfort, and longer hospital stay.

Conclusion: POUR is an important early postoperative complication in general surgical patients. Advanced age, male sex, diabetes mellitus, pre-existing lower urinary tract symptoms, spinal anesthesia, prolonged surgery, higher perioperative fluid administration, and postoperative opioid use were associated with increased risk. Careful preoperative risk assessment, early bladder monitoring, and timely intervention may help reduce catheter-related morbidity and support smoother postoperative recovery.

## Introduction

Postoperative urinary retention (POUR) is a common postoperative problem in which the patient is unable to pass urine despite bladder distension, often requiring intermittent or indwelling catheterization [1,2]. Though it may appear minor, it can cause suprapubic discomfort, anxiety, delayed mobilization, urinary tract infection, prolonged hospital stay, and, in severe cases, bladder overdistension with detrusor dysfunction [1-4]. As symptoms may be absent until the bladder is markedly full, active postoperative monitoring is important [5].

The reported incidence of POUR varies widely, from about 5% to 70%, mainly because of differences in definitions, surgical procedures, anesthetic techniques, fluid administration, analgesic use, and catheterization thresholds [1,3,6]. Some studies define POUR by failure to void within a fixed time, while others use bladder ultrasound volume, post-void residual urine, need for catheterization, or a combination of symptoms and objective findings [3,5,7]. This lack of uniform criteria often leads to inconsistent recognition and management in general surgical practice.

The mechanism of POUR is multifactorial. Normal voiding depends on detrusor contraction, urethral relaxation, intact autonomic and somatic innervation, bladder sensation, and voluntary control [1,2]. Surgery and anesthesia can disturb these mechanisms. Spinal and epidural anesthesia may temporarily block sacral parasympathetic pathways and reduce bladder sensation, while general anesthesia can suppress autonomic reflexes and delay the return of normal micturition [1,8]. Opioids increase urethral sphincter tone and reduce detrusor activity, whereas anticholinergics, sympathomimetics, and sedatives may further impair bladder emptying [1,2,9]. Pain, immobility, anxiety, and reluctance to void soon after surgery may also contribute.

Several patient-related factors increase the risk of POUR. Older age is a consistent predictor because of reduced detrusor function, associated comorbidities, and unrecognized lower urinary tract dysfunction [1,6,10]. Male sex and benign prostatic enlargement are important risk factors, especially after hernia, anorectal, and lower abdominal operations [1,7,11]. Diabetes mellitus may contribute through autonomic neuropathy and reduced bladder sensation [12]. Other reported associations include previous urinary retention, lower urinary tract symptoms, constipation, neurological disease, obesity, and use of drugs that affect bladder function [7,10,13]. Large surgical cohorts have also identified age, previous retention, constipation, anticholinergic use, longer operative duration, and bladder involvement in hernia as relevant predictors. 

The primary objective of this study was to determine the incidence of postoperative urinary retention among adult patients undergoing general surgical procedures. The secondary objectives were to identify the demographic, clinical, anesthetic, and perioperative factors associated with POUR and to assess its short-term outcomes, including catheterization requirement, repeated catheterization, urinary tract infection, delayed mobilization, catheter-related discomfort, and length of hospital stay.

Surgical and perioperative factors also play a major role. General surgical patients undergo procedures of varying complexity, from minor elective operations to prolonged emergency abdominal surgeries. Lower abdominal, pelvic, perineal, anorectal, and hernia surgeries carry a higher risk due to local pain, reflex inhibition of voiding, proximity to pelvic nerves, and difficulty increasing intra-abdominal pressure after surgery [11,14]. Longer operations increase exposure to anesthetic agents, intravenous fluids, and opioids, thereby increasing bladder filling and delaying voiding [6,7]. Excessive perioperative fluid administration may further increase bladder volume, especially when bladder sensation remains reduced after anesthesia [5,6].

POUR also affects early postoperative recovery. Catheterization gives immediate relief and prevents bladder injury, but it can cause pain, urethral trauma, hematuria, bacteriuria, urinary tract infection, reduced mobility, and patient dissatisfaction [4,15]. Delayed catheterization may lead to bladder overdistension, while unnecessary catheterization increases avoidable catheter-related complications [4,5,15]. Hence, a balanced approach that uses structured assessment, bladder scanning where available, and volume-based decisions for catheterization is preferred [5].

Although POUR is frequently encountered, it is not always studied separately in routine general surgical settings, particularly in resource-limited hospitals where bladder scanners may not be consistently available. Many studies have focused on orthopedic, gynecological, urological, hernia, or anorectal procedures, whereas fewer have evaluated POUR in mixed general surgical populations [6,7,11,14]. Such data are useful because they reflect real-world postoperative care with variable anesthetic methods, emergency procedures, different analgesic practices, and heterogeneous patient profiles.

Identifying high-risk patients early may help reduce postoperative morbidity. Simple variables such as age, sex, diabetes mellitus, lower urinary tract symptoms, type and duration of surgery, anesthetic technique, intraoperative fluid volume, and postoperative opioid use can be recorded easily and used to guide bladder monitoring. Preventive measures include avoiding unnecessary overhydration, encouraging early mobilization, limiting opioid use when possible, using bladder ultrasound where available, and applying clear catheterization criteria [4,5,16-18]. Alpha-blockers, such as tamsulosin, have been studied in selected surgical groups, but their routine use for all patients is not universally recommended because outcomes vary by procedure, sex, and baseline urinary risk [17-20].

The present study was therefore undertaken to assess the frequency of POUR among patients undergoing general surgical procedures and to identify patient-related, anesthetic, surgical, and postoperative factors associated with its occurrence. It also evaluated short-term outcomes, including catheterization requirement, repeated catheterization, urinary tract infection, and duration of hospital stay. By documenting these factors in a general surgery population, the study may help improve risk stratification and support practical postoperative bladder care.

## Materials and methods

Study design

This was a prospective observational study conducted among patients undergoing general surgical procedures. The primary objective was to determine the incidence of POUR during the early postoperative period. The secondary objectives were to identify the demographic, clinical, anesthetic, and perioperative factors associated with POUR and to assess its short-term outcomes, including catheterization requirement, repeated catheterization, urinary tract infection, delayed mobilization, catheter-related discomfort, and duration of hospital stay.

As this was an observational study, the routine surgical, anesthetic, and postoperative management of patients was not changed. All eligible patients were followed during the immediate postoperative period, and relevant clinical details were collected using a structured proforma. These data were used to assess the occurrence of POUR and to study the factors and short-term outcomes linked with its development.

Place of study

The study was carried out in the Department of General Surgery, Chalmeda Anand Rao Institute of Medical Sciences, Karimnagar, Telangana. Patients admitted under the Department of General Surgery for elective or emergency surgical procedures were screened for eligibility and enrolled based on the study criteria.

Study duration

The study was conducted over a period of two years, from April 2024 to March 2026. During this period, eligible patients undergoing general surgical procedures were included and monitored for the development of postoperative urinary retention and its short-term consequences.

Study population

The study population consisted of adult patients admitted for general surgical procedures at Chalmeda Anand Rao Institute of Medical Sciences during the study period. Both elective and emergency surgical cases were included to reflect the routine clinical workload of a general surgery department. Patients were observed during the early postoperative period to assess voiding status, need for catheterization, and associated clinical outcomes.

Sample size 

All patients satisfying the inclusion and exclusion criteria during the study period were considered for enrollment. The sample size was estimated using the standard formula n = Z²pq/d². In this formula, Z was taken as 1.96 at a 95% confidence level, p represented the expected proportion of postoperative urinary retention, *q* was calculated as 1−*p*, and *d* represented the allowable margin of error. Based on earlier reports, the anticipated incidence of postoperative urinary retention after surgical procedures was considered to be around 15%. With an absolute precision of 5%, the minimum sample size required was 196 patients. To compensate for possible exclusions or incomplete data, 220 eligible patients were finally included in the study. For analysis, the study population was divided into two groups: patients who developed POUR and patients who did not develop POUR.

The inclusion and exclusion criteria followed for patient enrollment are presented in Table 1.

**Table 1:** Inclusion and exclusion criteria.

S. no.	Inclusion criteria	Exclusion criteria
1	Patients aged 18 years and above	Patients with urinary retention before surgery
2	Patients undergoing elective or emergency general surgical procedures	Patients referred from outside with an indwelling urinary catheter already in place
3	Patients assessed preoperatively and admitted for postoperative monitoring	Patients with known neurogenic bladder
4	Patients undergoing surgery under spinal or general anaesthesia	Patients with urethral stricture or obstructive uropathy
5	Patients willing to provide informed consent	Patients with previous urinary tract surgery or pathology causing voiding difficulty

Definition of postoperative urinary retention

Postoperative urinary retention was defined as the inability to pass urine within six to eight hours after surgery, with associated suprapubic discomfort, lower abdominal fullness, palpable bladder distension, or a bladder volume significant enough to require catheterization. In patients where bedside bladder scan or ultrasonography was available, bladder volume measurement was used to support the diagnosis. In other patients, the diagnosis was made based on clinical assessment and the need for catheterization as decided by the treating team.

Preoperative assessment

All patients underwent routine preoperative evaluation according to institutional protocol. Demographic details, such as age, sex, body mass index, occupation, and relevant clinical history, were recorded. Particular attention was given to comorbid conditions, such as diabetes mellitus, hypertension, chronic kidney disease, neurological illness, and previous history of urinary retention. In male patients, symptoms suggestive of lower urinary tract obstruction, including hesitancy, poor urinary stream, nocturia, incomplete emptying, and increased frequency of micturition, were specifically noted.

Baseline investigations were performed as required for surgery and anesthesia fitness. These included complete blood count, renal function tests, blood glucose levels, urine routine examination, and other procedure-specific investigations. Any history of regular medication, especially drugs that could influence bladder function, such as anticholinergics, opioids, sedatives, antidepressants, or sympathomimetic agents, was also recorded.

Surgical and anesthetic details

Details of the surgical procedure were documented for each patient. These included the indication for surgery, whether the procedure was elective or emergency, the type of operation, the anatomical site of surgery, and the total duration of the procedure. For analysis, surgical procedures were broadly grouped into lower abdominal surgeries, upper abdominal surgeries, hernia-related procedures, anorectal procedures, soft-tissue procedures, and other general surgical procedures.

Anesthetic details were obtained from the anesthesia records. The type of anesthesia used, including general, spinal, epidural, or regional anesthesia, was recorded. Information regarding intraoperative medications, opioid administration, duration of anesthesia, and perioperative intravenous fluid volume was also collected. These factors were documented because anesthetic technique, analgesic use, and fluid administration can influence postoperative bladder function and the risk of urinary retention.

Postoperative monitoring

After surgery, patients were monitored in the recovery area and subsequently in the postoperative ward as per routine hospital practice. The time of completion of surgery and the time of first postoperative voiding were recorded. Patients were observed for symptoms such as inability to pass urine, suprapubic pain, restlessness, lower abdominal distension, and discomfort.

Patients who did not void within the expected postoperative period were clinically examined for bladder distension. When available, bladder ultrasonography or bedside bladder scan was used to estimate bladder volume. If urinary retention was suspected clinically or confirmed by bladder volume assessment, catheterization was performed according to the decision of the treating surgical team.

Catheterization protocol

Patients who developed postoperative urinary retention were managed by intermittent catheterization or temporary indwelling urinary catheterization depending on the clinical situation. The volume of urine drained at the time of catheterization was recorded wherever possible. Patients who required repeated catheterization, prolonged catheter drainage, or a failed trial of voiding after catheter removal were documented separately.

The decision regarding catheter removal was taken by the treating surgeon based on the patient’s recovery, mobility, pain status, and ability to void. After removal of the catheter, patients were observed for recurrence of urinary retention. Any catheter-related symptoms such as burning micturition, fever, hematuria, or discomfort were also recorded.

Variables studied

The main patient-related variables included age, sex, body mass index, diabetes mellitus, hypertension, neurological illness, history of lower urinary tract symptoms, previous urinary retention, and medication history. Surgery-related variables included type of procedure, elective or emergency nature of surgery, anatomical site, duration of surgery, and postoperative pain. Anesthesia-related variables included type of anesthesia, use of spinal or epidural technique, general anesthesia, opioid use, and volume of perioperative intravenous fluids.

Outcome variables included the development of postoperative urinary retention, need for catheterization, requirement for repeated catheterization, duration of catheter use, recurrence after catheter removal, urinary tract infection, delay in mobilization, and length of hospital stay. These variables were selected to assess both the occurrence of POUR and its immediate clinical impact.

Short-term outcomes assessed

The short-term outcomes assessed in this study included the need for single or repeated catheterization, the requirement for indwelling catheter placement, the duration of catheter use, the recurrence of retention after catheter removal, and the development of a urinary tract infection. The occurrence of catheter-related discomfort, delayed ambulation, increased postoperative pain or anxiety, and length of hospital stay were also recorded. These outcomes were assessed during the hospital stay and immediate postoperative follow-up period.

Ethical considerations

The study was conducted after obtaining approval from the Institutional Ethics Committee of Chalmeda Anand Rao Institute of Medical Sciences. Written informed consent was obtained from all participants before enrollment. Patient confidentiality was maintained throughout the study, and all data were used only for research purposes. Participation in the study did not interfere with standard treatment, and every patient received routine surgical, anesthetic, and postoperative care as advised by the treating team.

Data collection method

Data were collected using a predesigned study proforma. The proforma included demographic details, clinical history, comorbidities, preoperative assessment findings, surgical details, anesthetic details, postoperative voiding status, catheterization details, and short-term outcomes. Information was obtained from patient interviews, clinical examination, operative notes, anesthesia records, nursing records, and postoperative follow-up notes. The collected data were reviewed for completeness before analysis.

Statistical analysis

Data were first entered in Microsoft Excel (Microsoft Corporation, Redmond, Washington, USA) and then analyzed using IBM SPSS Statistics, version 30.0 (IBM Corp., Armonk, New York, USA). Continuous variables, such as age, operative duration, intraoperative fluid volume, and length of hospital stay, were summarized as the mean with standard deviation. Categorical variables, including sex, comorbidities, anesthetic technique, type of surgery, postoperative opioid use, and development of postoperative urinary retention, were presented as numbers and percentages.

Patients with POUR were compared with those without POUR to assess possible associated factors. The Chi-square test was used for categorical variables, while Fisher’s exact test was applied when the expected cell count was small. Continuous variables were compared using the independent t-test for normally distributed data and the Mann-Whitney U test for non-normally distributed data.

To adjust for possible confounding, multivariable logistic regression analysis was performed. Variables with clinical relevance and variables showing significant association in univariate analysis were entered into the model. These included age, sex, diabetes mellitus, lower urinary tract symptoms, type of surgery, anesthetic technique, operative duration, perioperative intravenous fluid volume, and postoperative opioid use. Adjusted odds ratios with 95% confidence intervals were calculated to identify independent predictors of POUR. A p-value <0.05 was considered statistically significant.

## Results

Study population and incidence of postoperative urinary retention (POUR)

**Figure 1:**
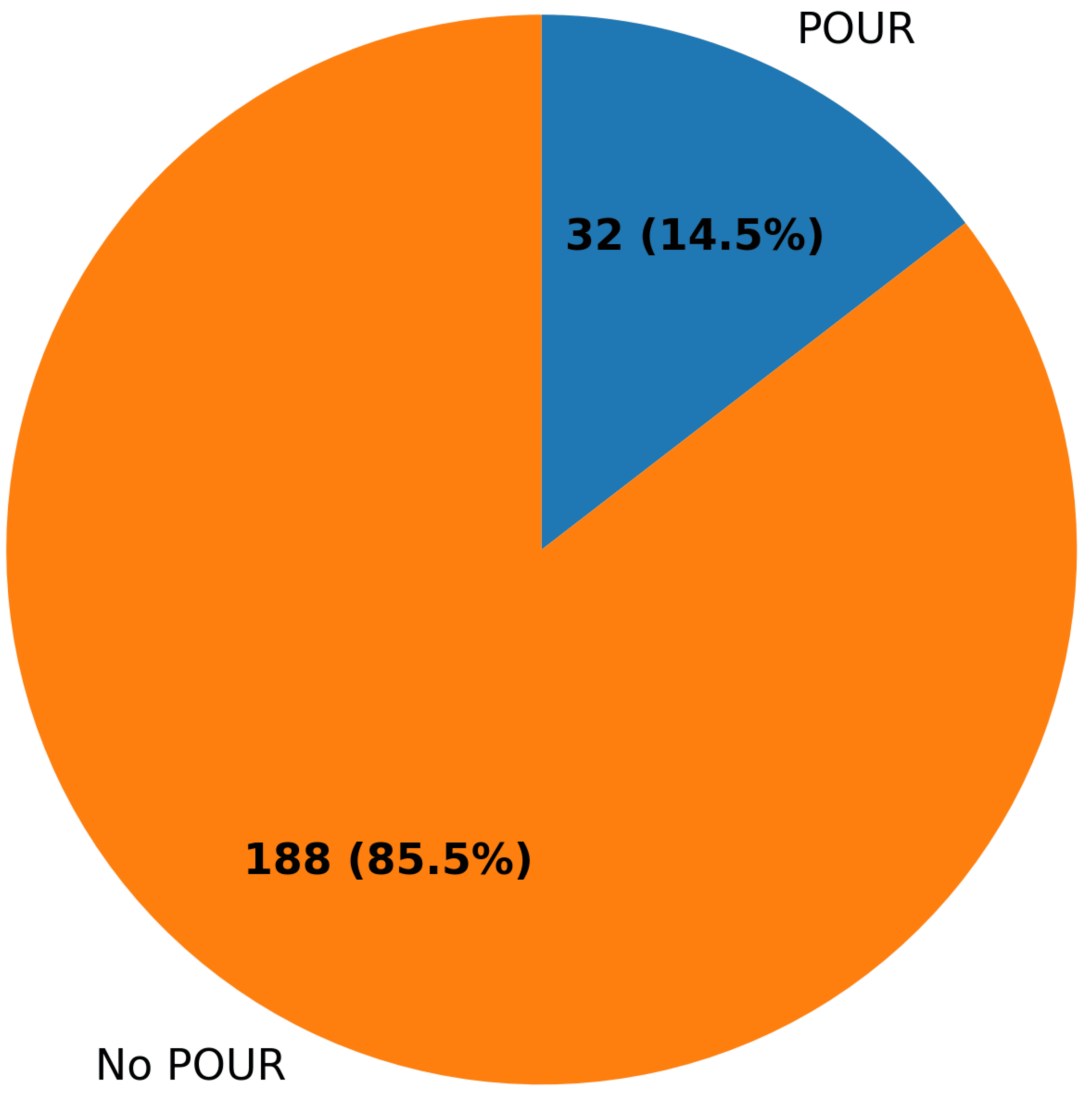
Incidence of POUR among the study population. POUR: postoperative urinary retention.

A total of 220 patients who underwent different general surgical procedures were included in the final analysis. The mean age of the study participants was 48.6 ± 14.8 years, and the age of the patients ranged from 18 to 78 years. The study population had almost equal representation of both sexes, with 112 males (50.9%) and 108 females (49.1%). During the postoperative monitoring period, postoperative urinary retention was identified in 32 patients (14.5%). The remaining 188 patients (85.5%) passed urine without retention and did not require bladder catheterization for POUR. Thus, most patients had an uneventful postoperative urinary outcome, while a smaller but clinically relevant proportion developed urinary retention after surgery. The overall distribution of patients with and without POUR is presented in Figure 1.

Association of demographic and clinical factors with postoperative urinary retention (POUR)

POUR varied according to selected demographic and clinical characteristics. Patients aged more than 60 years had a higher occurrence of POUR than those aged 60 years or below. Among patients above 60 years, POUR was observed in 16 of 63 patients (25.4%), whereas 16 of 157 patients (10.2%) aged 60 years or below developed POUR. This difference was statistically significant (χ²= 8.36, p = 0.004).

A similar pattern was noted with respect to sex. Among 112 male patients, 22 patients (19.6%) developed POUR, while among 108 female patients, 10 patients (9.3%) developed POUR. The association between sex and POUR was statistically significant (χ²= 4.77, p = 0.029). Diabetes mellitus also showed a significant association with POUR, with urinary retention occurring in 12 of 50 diabetic patients (24.0%) compared with 20 of 170 non-diabetic patients (11.8%) (χ² = 4.05, p = 0.03).

Lower urinary tract symptoms were strongly associated with postoperative urinary retention. POUR was documented in 10 of 30 patients (33.3%) with lower urinary tract symptoms, compared with 22 of 190 patients (11.6%) without such symptoms (χ²= 9.03, p = 0.002). In contrast, hypertension did not show a statistically significant association with POUR. These findings are summarized in Table 2.

**Table 2:** Association of demographic and clinical variables with POUR POUR: postoperative urinary retention. Values are expressed as number and percentage. χ² values are presented with degrees of freedom. For all two-category comparisons, df = 1. *A p-value <0.05 was considered statistically significant

Variable	Total patients, n	POUR present, n (%)	POUR absent, n (%)	Chi-square value	p-value
Age ≤60 years	157	16 (10.2)	141 (89.8)	8.36	0.004*
Age >60 years	63	16 (25.4)	47 (74.6)		
Male	112	22 (19.6)	90 (80.4)	4.77	0.029*
Female	108	10 (9.3)	98 (90.7)		
Diabetes mellitus present	50	12 (24.0)	38 (76.0)	4.65	0.031*
Diabetes mellitus absent	170	20 (11.8)	150 (88.2)		
Hypertension present	68	11 (16.2)	57 (83.8)	0.21	0.646
Hypertension absent	152	21 (13.8)	131 (86.2)		
Lower urinary tract symptoms present	30	10 (33.3)	20 (66.7)	9.86	0.002*
Lower urinary tract symptoms absent	190	22 (11.6)	168 (88.4)		

Surgical and anesthesia-related risk factors

Perioperative variables were compared between patients who developed postoperative urinary retention and those who did not. POUR was significantly more frequent among patients who received spinal anesthesia, underwent procedures lasting more than 90 minutes, received more than 1500 mL of perioperative intravenous fluids, and required postoperative opioid analgesia. The occurrence of POUR did not differ significantly between elective and emergency procedures. The association of perioperative and anesthesia-related factors with POUR is presented in Table 3.

**Table 3:** Association of perioperative factors with POUR. IV: intravenous; POUR: postoperative urinary retention. Values are expressed as number and percentage. Percentages were calculated using the total number of patients in the corresponding subgroup as the denominator. The chi-square test was used for two-category comparisons, with df = 1. *A p-value <0.05 was considered statistically significant.

Variable	Total patients, n	POUR present, n (%)	POUR absent, n (%)	Chi-square value	p-value
Elective surgery	150	20 (13.3)	130 (86.7)	0.56	0.455
Emergency surgery	70	12 (17.1)	58 (82.9)		
Spinal anesthesia	89	21 (23.6)	68 (76.4)	9.85	0.002*
General/regional anesthesia	131	11 (8.4)	120 (91.6)		
Surgery duration ≤90 minutes	155	15 (9.7)	140 (90.3)	10.00	0.002*
Surgery duration >90 minutes	65	17 (26.2)	48 (73.8)		
IV fluids ≤1500 mL	137	12 (8.8)	125 (91.2)	9.78	0.002*
IV fluids >1500 mL	83	20 (24.1)	63 (75.9)		
Postoperative opioid used	105	23 (21.9)	82 (78.1)	8.75	0.003*
No postoperative opioid	115	9 (7.8)	106 (92.2)		

**Figure 2:**
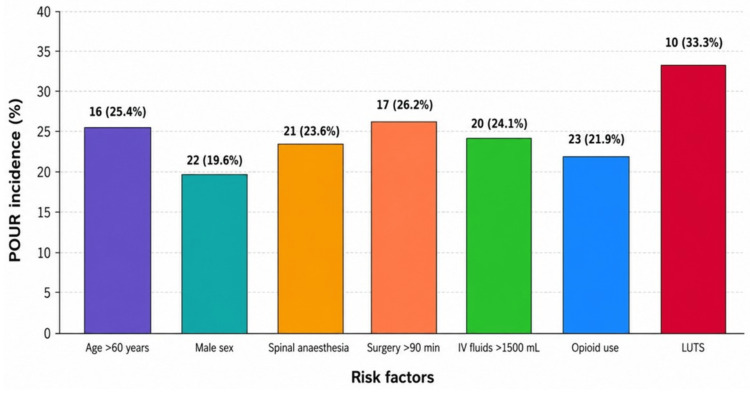
Incidence of POUR across important risk factors. POUR: postoperative urinary retention; LUTS: lower urinary tract symptoms; IV: intravenous.

Patients receiving spinal anesthesia had a higher occurrence of POUR than those receiving general or regional anesthesia (21/89 (23.6%) vs 11/131 (8.4%); chi-square = 9.85, p = 0.002). Operative duration also showed a significant association, with POUR occurring in 17/65 patients (26.2%) when surgery lasted more than 90 minutes, compared with 15/155 patients (9.7%) when the duration was 90 minutes or less (chi-square = 10.00, p = 0.002). A similar pattern was observed with perioperative fluid administration, where patients receiving more than 1500 mL had a higher frequency of POUR (20/83 (24.1%)) than those receiving 1500 mL or less (12/137 (8.8%); chi-square = 9.78, p = 0.002). Postoperative opioid use was also significantly associated with POUR (23/105 (21.9%) vs 9/115 (7.8%); chi-square = 8.75, p = 0.003). In contrast, the difference between elective and emergency surgery was not statistically significant. The distribution of POUR across the major perioperative risk factors is shown in Figure 2.

Distribution of postoperative urinary retention (POUR) according to type of surgery

The frequency of postoperative urinary retention varied according to the type of surgical procedure. The highest number of POUR cases was seen among patients undergoing hernia-related procedures, followed by lower abdominal surgeries and anorectal procedures. POUR occurred in 12 out of 54 patients (22.2%) undergoing hernia-related surgery and 10 out of 48 patients (20.8%) undergoing lower abdominal surgery. Anorectal procedures showed POUR in 6 out of 38 patients (15.8%) (Table 4).

**Table 4:** Occurrence of POUR according to type of general surgical procedure. POUR: postoperative urinary retention. Chi-square test for overall comparison: χ² = 10.07, p = 0.073. χ² values are reported with degrees of freedom. For two-category comparisons, df = 1. A p-value <0.05 was considered statistically significant.

Type of surgery	Total patients, n	POUR present, n (%)	POUR absent, n (%)
Hernia-related procedures	54	12 (22.2)	42 (77.8)
Lower abdominal procedures	48	10 (20.8)	38 (79.2)
Anorectal procedures	38	6 (15.8)	32 (84.2)
Upper abdominal procedures	34	2 (5.9)	32 (94.1)
Soft tissue procedures	28	1 (3.6)	27 (96.4)
Other procedures	18	1 (5.6)	17 (94.4)
Total	220	32 (14.5)	188 (85.5)

**Figure 3:**
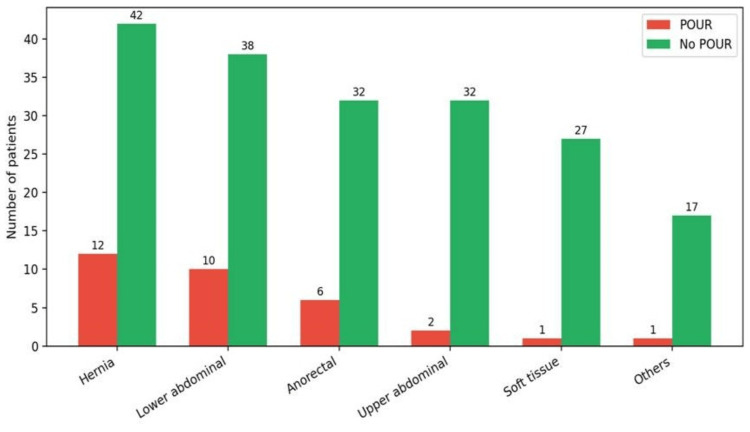
POUR according to type of general surgical procedure. POUR: postoperative urinary retention.

Subgroup analysis according to type of surgery showed that POUR was more frequent after hernia-related procedures and lower abdominal surgeries than after upper abdominal, soft tissue, and other procedures. However, the overall association between procedure type and POUR was not statistically significant (χ² = 10.07, p = 0.073). Procedure type was also included in the multivariable logistic regression model to adjust for possible procedure-related variation. After adjustment for demographic and perioperative factors, procedure type did not remain an independent predictor of POUR (Figure 3).

Catheterization pattern among patients with postoperative urinary retention (POUR)

Among the 32 patients (14.5%) who developed postoperative urinary retention, most responded to initial bladder decompression. Single catheterization was sufficient in 25 patients (78.1%), and these patients were able to void subsequently without further intervention. Repeated catheterization was required in seven patients (21.9%) because they were unable to pass urine after the first catheterization attempt. Temporary indwelling catheter placement was needed in nine patients (28.1%). This was observed more often among elderly male patients, those with pre-existing lower urinary tract symptoms, and patients who had recurrent retention after catheter removal (Table 5).

**Table 5:** Catheterization pattern among patients who developed POUR. POUR: postoperative urinary retention. Percentages were calculated among patients who developed POUR.

Catheterization variable	Number of patients (n = 32)	Percentage
Single catheterization only	25	78.1
Repeated catheterization	7	21.9
Temporary indwelling catheter required	9	28.1
Successful voiding after catheter removal	29	90.6
Recurrent retention after catheter removal	3	9.4

The mean urine volume drained during the first catheterization was 620 ± 180 mL. Most patients passed urine successfully after catheter removal within 24 to 48 hours. None of the patients required emergency urological intervention during the immediate postoperative period.

Short-term postoperative outcomes

Patients who developed POUR showed a comparatively slower early postoperative recovery than those who did not develop retention. The mean hospital stay was longer among patients with POUR, with an average duration of 4.8 ± 1.6 days, compared with 3.2 ± 1.1 days among patients without POUR. This difference was statistically significant (p < 0.001), suggesting that urinary retention was associated with prolonged postoperative hospitalization (Table 6).

**Table 6:** Comparison of short-term outcomes between patients with and without POUR. Values are expressed as mean ± SD or number and percentage. POUR: postoperative urinary retention. Chi-square test was used for categorical variables and independent t-test was used for continuous variables. χ² values are reported with degrees of freedom. For two-category comparisons, df = 1. *A p-value <0.05 was considered statistically significant.

Outcome	POUR present (n = 32)	POUR absent (n = 188)	Test value	p-value
Mean hospital stay, days	4.8 ± 1.6	3.2 ± 1.1	t = 7.12	<0.001*
Urinary tract infection	5 (15.6)	6 (3.2)	χ² = 8.24	0.004*
Delayed mobilization	11 (34.4)	28 (14.9)	χ² = 7.01	0.008*
Repeated catheterization	7 (21.9)	0 (0.0)	χ² = 43.09	<0.001*
Catheter-related discomfort	8 (25.0)	0 (0.0)	χ² = 50.30	<0.001*

**Figure 4:**
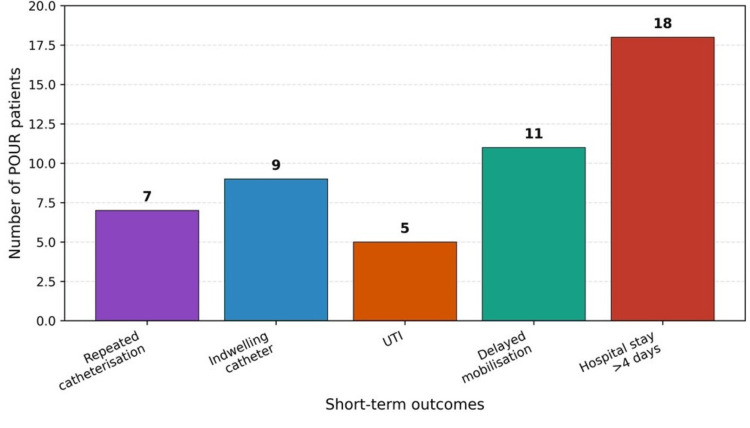
Short-term outcomes among patients with POUR. POUR: postoperative urinary retention; UTI: urinary tract infection.

Postoperative complications and recovery-related outcomes were less favorable among patients who developed POUR. Urinary tract infection was documented in five of 32 patients (15.6%) with POUR, compared with six of 188 patients (3.2%) without POUR. Delayed mobilization was also more frequent in the POUR group, occurring in 11 of 32 patients (34.4%), whereas it was observed in 28 of 188 patients (14.9%) among those without urinary retention. In addition, patients with POUR experienced greater postoperative discomfort, mainly due to bladder distension, catheterization, and catheter-related symptoms (Figure 4).

Multivariable logistic regression analysis

Multivariable logistic regression analysis was carried out to determine the independent predictors of postoperative urinary retention after adjusting for other relevant clinical and perioperative factors. Variables included in the model were age above 60 years, male sex, diabetes mellitus, lower urinary tract symptoms, spinal anesthesia, operative duration more than 90 minutes, perioperative intravenous fluid administration more than 1500 mL, and postoperative opioid use.

After adjustment, lower urinary tract symptoms, spinal anesthesia, operative duration more than 90 minutes, and perioperative intravenous fluid administration more than 1500 mL remained independently associated with postoperative urinary retention. Age above 60 years, male sex, diabetes mellitus, and postoperative opioid use showed higher odds, but these variables did not remain statistically significant in the adjusted model (Table 7).

**Table 7:** Multivariable logistic regression analysis for independent predictors of POUR. Values are expressed as adjusted odds ratio with 95% confidence interval. Variables that were clinically relevant and/or significant on univariate analysis were entered into the multivariable logistic regression model. *p-value <0.05 was considered statistically significant. IV: intravenous; POUR: postoperative urinary retention.

Variable entered in the model	Adjusted odds ratio	95% confidence interval	p-value
Age >60 years	1.72	0.72-4.11	0.218
Male sex	1.64	0.67-4.03	0.281
Diabetes mellitus	1.49	0.59-3.77	0.397
Lower urinary tract symptoms	3.08	1.10-8.61	0.032*
Spinal anaesthesia	2.71	1.10-6.68	0.030*
Operative duration >90 minutes	2.54	1.04-6.22	0.041*
IV fluids >1500 mL	2.42	1.00-5.86	0.049*
Postoperative opioid use	1.95	0.78-4.88	0.154

## Discussion

POUR is a recognized early complication after surgery, but it may be missed unless bladder function is actively observed. In the present study, POUR was seen in 32 of 220 patients, giving an incidence of 14.5%. This shows that nearly one in seven patients had difficulty in passing urine during the early postoperative period. The observed rate is within the range reported in earlier studies, although published figures vary widely because of differences in surgical procedures, anesthetic techniques, diagnostic criteria, timing of assessment, and catheterization thresholds [1-3]. Since this was an observational study, the findings should be interpreted as associations and not as proof of causation.

Increasing age was associated with a higher frequency of POUR. Patients aged above 60 years developed retention more often than younger patients. Similar observations have been reported in previous studies, where advancing age has been identified as an important risk factor for postoperative retention [1,6,10]. This may be related to reduced detrusor function, impaired bladder sensation, slower mobilization, associated comorbidities, and use of medications that can affect bladder emptying. In some elderly patients, mild pre-existing voiding difficulty may become clinically evident only after surgery and anesthesia.

Male patients had a higher occurrence of POUR than female patients, which is in agreement with earlier reports [1,7,10]. This association may be partly explained by unrecognized benign prostatic enlargement, bladder outlet obstruction, or pre-existing lower urinary tract symptoms. After surgery, pain, reduced mobility, anesthetic effects, and perioperative fluid administration may further increase the chance of retention in susceptible male patients.

Diabetes mellitus was also associated with increased occurrence of POUR. Diabetic patients may have reduced bladder sensation and impaired detrusor contraction due to autonomic neuropathy, even when urinary symptoms are not prominent before surgery [12]. This finding suggests that diabetic patients, particularly elderly patients and those undergoing longer procedures, may need closer postoperative voiding assessment.

Pre-existing lower urinary tract symptoms showed a strong association with POUR. Symptoms, such as hesitancy, poor stream, nocturia, increased frequency, and incomplete emptying, are easy to identify during preoperative history-taking. Earlier studies have also reported that urinary symptoms or previous retention increase the risk of POUR [7,10,13]. In the present study, lower urinary tract symptoms also remained an important factor after adjusted analysis, suggesting that baseline voiding difficulty has practical value in identifying patients who may require closer monitoring.

Anesthetic technique was another important factor. Patients receiving spinal anesthesia had a higher occurrence of POUR than those receiving general or regional anesthesia. Spinal anesthesia can temporarily affect sacral parasympathetic pathways, reduce bladder sensation, and delay recovery of detrusor activity [1,8]. As a result, the bladder may continue to fill even when the patient does not feel a strong urge to void. This supports careful observation of voiding status in patients who receive spinal anesthesia.

Operative duration was associated with POUR in this study. Patients whose procedures lasted more than 90 minutes had a higher frequency of retention. Longer procedures may increase exposure to anesthetic drugs, intravenous fluids, postoperative pain, and analgesic requirements, all of which can interfere with normal micturition [6,7,10]. Similarly, patients receiving more than 1500 mL of perioperative intravenous fluids had a higher occurrence of POUR. Higher fluid administration may lead to faster bladder filling during a period when bladder sensation and detrusor function are still recovering [5,6].

Postoperative opioid use was also associated with urinary retention on univariate analysis. Opioids can reduce detrusor contractility and increase urethral sphincter tone, thereby making bladder emptying more difficult [1,2,9]. However, adequate analgesia remains essential after surgery. Therefore, these findings should be viewed as supportive of balanced analgesic strategies and careful monitoring in patients who require opioid-based pain control, rather than as evidence to avoid opioids when clinically indicated.

Procedure type was analyzed because different general surgical procedures may carry different risks of POUR. In the present study, POUR was numerically more frequent after hernia-related, lower abdominal, and anorectal procedures. This pattern is clinically understandable, as local pain, perineal discomfort, reflex sphincter spasm, and difficulty in voiding posture may contribute to retention after these operations [10,11,14]. However, the overall association between procedure category and POUR did not reach statistical significance, and procedure type did not remain an independent predictor after adjustment for other factors. This may be due to overlap with operative duration, anesthetic technique, fluid volume, and analgesic use. The relatively small number of patients in some procedure subgroups may also have limited the strength of procedure-specific analysis.

Most patients who developed POUR improved after a single catheterization, while a smaller proportion required repeated catheterization or temporary indwelling catheter placement. This finding suggests that early recognition and timely bladder decompression can help avoid persistent bladder dysfunction. At the same time, catheterization is not free from harm and may cause discomfort, urethral trauma, bacteriuria, and urinary tract infection [4,15]. Hence, catheter use should be based on clinical symptoms, bladder distension, time since surgery, and bladder volume assessment wherever available.

Patients with POUR had less favorable short-term outcomes than those without retention. They had longer hospital stays, higher urinary tract infection rates, delayed mobilization, and catheter-related discomfort. These findings indicate that POUR is not merely a transient voiding problem but a complication that can affect early recovery and patient comfort [4,5,15]. The longer hospital stay may be related to catheter care, trial of voiding, treatment of urinary symptoms, and observation for recurrent retention.

The study adds practice-based information from a mixed general surgery cohort. Many of the identified risk factors are already known, but their evaluation in a heterogeneous routine surgical population is useful because postoperative bladder monitoring may vary across centers. The findings support the need for awareness of readily identifiable risk factors such as age, sex, diabetes, lower urinary tract symptoms, anesthetic technique, operative duration, fluid volume, and opioid use. However, as the study was observational and conducted at a single center, the results should not be interpreted as definitive recommendations. They may help guide risk awareness, careful postoperative observation, and timely bladder assessment in patients who appear more vulnerable to POUR [5].

Limitations

This study has a few limitations. It was conducted at a single center, so the findings may not fully represent practices in other hospitals. The study also included a mixed group of general surgical procedures. Although this reflects routine surgical practice, the number of patients in some procedure categories was small, which limited the strength of procedure-wise subgroup analysis. Procedure type was considered during analysis, but larger studies with adequate numbers in each surgical subgroup are needed to define the independent risk carried by specific operations more clearly.

Bladder volume assessment was not uniform for all patients because bedside bladder scan or ultrasonography was not available in every case. Therefore, in some patients, POUR was diagnosed mainly on the basis of clinical findings and the need for catheterization. The study assessed only short-term in-hospital outcomes, and long-term follow-up after discharge was not performed. Future multicenter studies with larger samples, standard bladder scan-based diagnostic criteria, and well-powered procedure-specific analysis may provide stronger evidence for developing practical prevention and management protocols for POUR.

## Conclusions

POUR was a relevant early complication among patients undergoing general surgical procedures. It was observed more often in older patients, males, diabetics, patients with pre-existing lower urinary tract symptoms, those receiving spinal anesthesia, patients undergoing longer operations, those receiving higher perioperative intravenous fluids, and those requiring postoperative opioid analgesia. Most cases were managed successfully with prompt bladder decompression and supportive care. However, POUR was associated with added postoperative problems, including repeated catheterization, urinary tract infection, delayed mobilization, catheter-related discomfort, and longer hospital stay. These findings support the need for early recognition of high-risk patients, careful postoperative bladder monitoring, and timely intervention to improve recovery after general surgical procedures.

## References

[1] Baldini G, Bagry H, Aprikian A, Carli F (2009). Postoperative urinary retention: anesthetic and perioperative considerations. Anesthesiology.

[2] Darrah DM, Griebling TL, Silverstein JH (2009). Postoperative urinary retention. Anesthesiol Clin.

[3] Agrawal K, Majhi S, Garg R (2019). Post-operative urinary retention: review of literature. World J Anesthesiol.

[4] Jackson J, Davies P, Leggett N (2019). Systematic review of interventions for the prevention and treatment of postoperative urinary retention. BJS Open.

[5] Chrouser K, Fowler KE, Mann JD (2024). Urinary retention evaluation and catheterization algorithm for adult inpatients. JAMA Netw Open.

[6] Kowalik U, Plante MK (2016). Urinary retention in surgical patients. Surg Clin North Am.

[7] Brouwer TA, van Roon EN, Rosier PF, Kalkman CJ, Veeger N (2021). Postoperative urinary retention: risk factors, bladder filling rate and time to catheterization: an observational study as part of a randomized controlled trial. Perioper Med (Lond).

[8] Niazi AA, Taha AM, Mowafi HA (2015). Postoperative urinary retention after general and spinal anesthesia in orthopedic surgical patients. Egypt J Anaesth.

[9] Bash LD, Turzhitsky V, Mark RJ, Hofer IS, Weingarten TN (2024). Post-operative urinary retention is impacted by neuromuscular block reversal agent choice: a retrospective cohort study in US hospital setting. J Clin Anesth.

[10] Croghan SM, Mohan HM, Breen KJ (2023). Global incidence and risk factors associated with postoperative urinary retention following elective inguinal hernia repair: the retention of urine after inguinal hernia elective repair (RETAINER I) study. JAMA Surg.

[11] Liu B, Chen Y, Zhang P, Long W, He H, Li X, Wang R (2024). Risk factors for postoperative urinary retention in patients underwent surgery for benign anorectal diseases: a nested case-control study. BMC Anesthesiol.

[12] Sung KH, Lee KM, Chung CY, Kwon SS, Lee SY, Ban YS, Park MS (2015). What are the risk factors associated with urinary retention after orthopaedic surgery?. Biomed Res Int.

[13] Köseoğlu E, Acar Ö, Kılıç M (2022). Urinary retention after non-urological surgeries: management patterns and predictors of prognosis. Continence.

[14] Toyonaga T, Matsushima M, Sogawa N (2006). Postoperative urinary retention after surgery for benign anorectal disease: potential risk factors and strategy for prevention. Int J Colorectal Dis.

[15] Meddings J, Rogers MA, Krein SL, Fakih MG, Olmsted RN, Saint S (2014). Reducing unnecessary urinary catheter use and other strategies to prevent catheter-associated urinary tract infection: an integrative review. BMJ Qual Saf.

[16] Sirisreetreerux P, Wattanayingcharoenchai R, Rattanasiri S, Pattanaprateep O, Numthavaj P, Thakkinstian A (2021). Medical and non-medical interventions for post-operative urinary retention prevention: network meta-analysis and risk-benefit analysis. Ther Adv Urol.

[17] Li H, Zhang W, Xu G (2022). Prophylactic tamsulosin can reduce the risk of urinary retention after surgery in male patients: a systematic review and meta-analysis. Front Surg.

[18] Gao B, Zhang D, Wang Y, Wang Z, Wang Z (2023). The effect of tamsulosin in postoperative urinary retention: a meta-analysis of randomized controlled trials. Naunyn Schmiedebergs Arch Pharmacol.

[19] Chen Q, Li N, Wu Y (2024). Neostigmine for postoperative surgical urine retention: a systematic review and meta-analysis. Adv Clin Exp Med.

[20] Cambise C, De Cicco R, Luca E (2024). Postoperative urinary retention (POUR): a narrative review. Saudi J Anaesth.

